# Peer Review for “Applications of Indocyanine Green in Breast Cancer for Sentinel Lymph Node Mapping: Protocol for a Scoping Review”

**DOI:** 10.2196/69705

**Published:** 2025-01-06

**Authors:** Anonymous

**Keywords:** indocyanine green, ICG, sentinel lymph node, breast cancer, breast, fluorescence, axillary lymph node mapping, NIR, surgical planning, near-infrared

*This is the peer-review report for “Applications of Indocyanine Green in Breast Cancer for Sentinel Lymph Node Mapping: Protocol for a Scoping Review.”*

## Round 1 Review

### General Comments

This paper [1] summarized the application value and existing problems of indocyanine green (ICG) in sentinel lymph node (SLN) biopsy of early breast cancer, which has positive significance for improving the accuracy of clinical SLN detection. This study has certain clinical value.

### Specific Comments

#### Major Comments

1. Due to the high hardware requirements for the clinical application of ICG, the number of relevant studies in the search is relatively small. It is hoped that the author can search the recent relevant literature to improve the credibility of this review.
2. It is hoped that the author will analyze and compare the advantages and disadvantages of ICG and traditional SLN biopsy methods, so as to guide clinicians to adopt appropriate methods for appropriate patients.

## Abbreviations

ICG: indocyanine green
SLN: sentinel lymph node
